# The Endothelial Glycocalyx in Pig-to-Baboon Cardiac Xenotransplantation—First Insights

**DOI:** 10.3390/biomedicines12061336

**Published:** 2024-06-16

**Authors:** Martin Bender, Jan-Michael Abicht, Bruno Reichart, Maria Leuschen, Felicia Wall, Julia Radan, Elisabeth Neumann, Maren Mokelke, Ines Buttgereit, Sebastian Michel, Reinhard Ellgass, Katja Gieseke, Stig Steen, Audrius Paskevicius, Joachim Denner, Antonia W. Godehardt, Ralf R. Tönjes, Christian Hagl, David Ayares, Eckhard Wolf, Michael Schmoeckel, Paolo Brenner, Martin B. Müller, Matthias Längin

**Affiliations:** 1 Department of Anaesthesiology, University Hospital, LMU Munich, 81377 Munich, Germany; 2 Transregional Collaborative Research Center 127, Walter Brendel Centre of Experimental Medicine, LMU Munich, 81377 Munich, Germany; 3 Department of Cardiac Surgery, University Hospital, LMU Munich, 81377 Munich, Germany; 4 Munich Heart Alliance, German Center for Cardiovascular Research (DZHK), 81377 Munich, Germany; 5 Department of Cardiothoracic Surgery, Lund University and Skåne University Hospital, 221 85 Lund, Sweden; 6 Institute of Virology, Free University Berlin, 14163 Berlin, Germany; 7 Division of Haematology, Cell and Gene Therapy, Paul-Ehrlich-Institut, 63225 Langen, Germany; 8 Revivicor, Blacksburg, VA 24060, USA; 9 Institute of Molecular Animal Breeding and Biotechnology, Gene Center, and Department of Veterinary Sciences, LMU Munich, 81377 Munich, Germany; 10 Center for Innovative Medical Models (CiMM), LMU Munich, 81377 Munich, Germany; 11 Interfaculty Center for Endocrine and Cardiovascular Disease Network Modelling and Clinical Transfer (ICONLMU), LMU Munich, 81377 Munich, Germany

**Keywords:** heart, xenotransplantation, endothelial glycocalyx, endothelial activation, organ preservation, orthotopic heart transplantation

## Abstract

Cardiac xenotransplantation has seen remarkable success in recent years and is emerging as the most promising alternative to human cardiac allotransplantation. Despite these achievements, acute vascular rejection still presents a challenge for long-term xenograft acceptance and new insights into innate and adaptive immune responses as well as detailed characterizations of signaling pathways are necessary. In allotransplantation, endothelial cells and their sugar-rich surface—the endothelial glycocalyx—are known to influence organ rejection. In xenotransplantation, however, only in vitro data exist on the role of the endothelial glycocalyx so far. Thus, in the current study, we analyzed the changes of the endothelial glycocalyx components hyaluronan, heparan sulfate and syndecan-1 after pig-to-baboon cardiac xenotransplantations in the perioperative (n = 4) and postoperative (n = 5) periods. These analyses provide first insights into changes of the endothelial glycocalyx after pig-to-baboon cardiac xenotransplantation and show that damage to the endothelial glycocalyx seems to be comparable or even less pronounced than in similar human settings when current strategies of cardiac xenotransplantation are applied. At the same time, data from the experiments where current strategies, like non-ischemic preservation, growth inhibition or porcine cytomegalovirus (a porcine roseolovirus (PCMV/PRV)) elimination could not be applied indicate that damage of the endothelial glycocalyx also plays an important role in cardiac xenotransplantation.

## 1. Introduction

Cardiac xenotransplantation has seen remarkable success in recent years and is emerging as the most promising alternative to human cardiac allotransplantation [1,2,3]. This success was made possible by essential achievements and important findings in different in vivo and in vitro models [4,5,6]: the development of genetically modified donor pigs lacking surface sugar antigens as well as expressing different human genes [1,7], immunosuppression based on co-stimulation blockade of the CD40/CD40 ligand (CD40L) pathway [8], continuous cold non-ischemic heart preservation [9,10], the relevance of growth control [4,11,12,13], the absence of porcine cytomegalovirus (a porcine roseolovirus (PCMV/PRV)) [14,15] and other pathogens in the donor animals [1,16] and the relevant role of inflammatory responses and coagulation disorders following xenotransplantation [17,18,19,20]. Using these current strategies, consistent survival for up to nine months was achieved in life-supporting pig-to-baboon experiments [4,5,6] and the first pig-to-human cardiac xenotransplantations were performed as individual medical treatments in 2022 [21,22] and 2023 [23].

Despite these outstanding achievements, acute vascular rejection still presents a challenge for long-term xenograft acceptance [24] and new insights into innate and adaptive immune responses as well as detailed characterizations of signaling pathways are necessary. In our pig-to-baboon xenotransplantation experience, attempts to reverse acute vascular rejection using high-dose steroid therapy have proven uniformly unsuccessful [1]. Therefore, we suggest that attention should be directed to safely preventing acute vascular rejection as well as developing and testing potential treatment options in pre-clinical models [1].

Endothelial dysfunction and persistent endothelial inflammation are known as hallmarks of acute vascular rejection in allogenic transplantation [25]. Endothelial health is closely linked to an intact endothelial sugar-rich surface layer known as the endothelial glycocalyx [26], while endothelial dysfunction is not only accompanied but also reinforced by the degradation of the endothelial glycocalyx [27]. It is mainly composed of core proteoglycans from the syndecan and glypican families, carrying highly sulfated, linear glycosaminoglycan attachments, including heparan sulfate and chondroitin sulfate, as well as non-sulfated receptor-bound hyaluronan [28,29]. Endothelial glycocalyx integrity plays a major role in preventing acute vascular rejection by limiting inflammation and maintaining endothelial homeostasis [30]. In human liver [31], lung [32], and kidney [33] allotransplantation, damage to the endothelial glycocalyx, indicated by elevated plasma concentrations of glycocalyx breakdown products, is correlated with reduced organ survival and early graft rejection. In xenotransplantation, however, only in vitro data exist on the role of the endothelial glycocalyx so far [34,35,36], indicating that glycocalyx shedding is linked to complement activation and xenograft rejection [35,36], while protection against complement activation contributes to maintaining an intact glycocalyx layer on endothelial cells [34].

To broaden these initial in vitro findings and to gain further insights into the role of the endothelial glycocalyx in cardiac xenotransplantation, we retrospectively analyzed the changes in the plasma concentrations of the major endothelial glycocalyx components hyaluronan, heparan sulfate and syndecan-1 as surrogate biomarkers for glycocalyx integrity following pig-to-baboon cardiac xenotransplantation.

## 2. Materials and Methods

### 2.1. Animals and Study Groups

Hearts from five genetically modified piglets were transplanted into male baboons. The piglets (German Landrace/Large White; blood group 0) were homozygous for alpha1,3-galactosyltransferase knockout (GGTA1-KO) and hemizygous transgenic for human CD46 (hCD46) and human thrombomodulin (hTBM) (Revivicor, Blacksburg, VA, USA and Institute of Molecular Animal Breeding and Biotechnology, Gene Center, LMU Munich, Munich, Germany). Five baboons (*Papio anubis* and *Papio hamadryas*; blood group B; German Primate Centre (DPZ), Göttingen, Germany) served as recipients. Expression of hCD46 and hTBM was verified post mortem by immunohistochemistry. Two animals were tested positive for PCMV/PRV, as published elsewhere [5,14].

Four baboons, #17186, #17290, #17494 and #17492, were analyzed in the perioperative and postoperative period after pig-to-baboon cardiac xenotransplantation. These studies were divided into two groups in the postoperative analyses: those that were deliberately terminated after 90 postoperative days (#17186 and #17290, group I) and animals that were tested positive for PCMV/PRV (#17494 and #17492, group II). As probes were available for the postoperative period (but perioperative probes were no longer available), a fifth baboon, #16755, was analyzed in this period. In this animal, the xenograft was ischemically preserved and no growth-inhibiting drugs were administered (see below), as described in detail elsewhere [4].

The study was approved by the Government of Upper Bavaria. All animals were cared for and treated in accordance with the Guide for the Care and Use of Laboratory Animals (German Legislation for the Welfare of Laboratory Animals and US National Institutes of Health).

### 2.2. Anesthesia, Surgical Procedure and Heart Preservation

After sedation, induction of anesthesia and endotracheal intubation of the animals [37], surgery was conducted as published in detail elsewhere [4].

In brief, after median sternotomy of the donor animal, the aorta was cross-clamped. In one animal, #16755, the heart was perfused with a single dose of 20 mL/kg crystalloid Belzer’s UW cardioplegic solution (Preservation Solutions, Elkhorn, WI, USA) at 4 °C. The appendices of the right and left atrium were opened for decompression. The heart was then excised, submersed in cardioplegic solution and stored on ice. In four animals, #17186, #17290, #17494 and #17492, antegrade non-ischemic preservation commenced immediately after cross-clamping of the aorta; continuous perfusion with 8 °C oxygenated, hyperoncotic solution containing albumin, hormones, nutrients and erythrocytes [9,10] was provided by an extracorporeal heart preservation system (University of Lund, Sweden) consisting of a pressure- and flow-controlled roller pump, an O_2_/CO_2_ exchanger, a leukocyte filter and a cooler/heater unit. During storage, the heart was preserved the same way and the perfusion pressure kept at 20 mmHg.

After median sternotomy in the baboon recipient, extracorporeal circulation was installed and started. Explantation of the recipient’s native heart and xenotransplantation followed the techniques of Lower and Shumway [38]. In the four animals with non-ischemic preservation, the donor heart was intermittently perfused for 2 min every 15 min during implantation, as described in detail elsewhere [10].

### 2.3. Immunosuppression, Anti-Inflammatory and Additive Therapy

Immunosuppressive therapy was based on a CD40/CD40L co-stimulation blockade [8]. For induction therapy, all animals received B cell depleting anti-CD20 ab (Mabthera; Roche Pharma, Basel, Switzerland), anti-thymocyte globulin (ATG, thymoglobulin; Sanofi, Paris, France) and a mouse/rhesus chimeric anti-CD40 IgG4 monoclonal antibody (anti-CD40 Mab; 50 mg/kg body weight (bw); mouse/rhesus chimeric IgG4 clone 2C10R4, NIH Non-human Primate Reagent Resource; Mass Biologicals, Boston, MA, USA; courtesy of K. Reimann).

Immunosuppression was maintained with mycophenolate mofetil (CellCept; Roche Pharma, Basel, Switzerland), methylprednisolone (urbasone soluble; Sanofi, Paris, France) and anti-CD40 Mab (50 mg/kg bw once weekly).

All animals received anti-inflammatory therapy including an C1 esterase inhibitor (Berinert; CSL Behring, King of Prussia, PA, USA), an interleukin 6 (IL-6) receptor antagonist (RoActemra; Roche Pharma, Basel, Switzerland), a TNFα inhibitor (Enbrel; Pfizer, New York, NY, USA) and an IL-1 receptor antagonist (Kineret; Swedish Orphan Biovitrum, Solna, Sweden) [4,19].

The additive medication consisted of acetylsalicylic acid (Aspirin; Bayer, Leverkusen, Germany) and unfractionated heparin (Heparin-Natrium-25000-ratiopharm^®^; Ratiopharm, Ulm, Germany). Furthermore, ganciclovir (Cymevene, Roche Pharma, Basel, Switzerland), cefuroxime (Cefuroxim; Hikma Pharmaceuticals, London, UK) and epoetin beta (NeoRecormon 5000; Roche Pharma, Basel, Switzerland) were also administered [4].

Four animals, #17186, #17290, #17494 and #17492, received a therapeutic regime to slow xenograft overgrowth, which was described in detail elsewhere [4,5]. Methylprednisolone was tapered down quickly and additional antihypertensive drugs (enalapril (Enahexal; Hexal, Holzkirchen, Germany) and metoprolol tartrate (Beloc; AstraZeneca, Cambridge, UK)) as well as the mTOR inhibitor temsirolimus (Torisel; Pfizer, New York, NY, USA) were added.

### 2.4. Blood Sampling and Lactate Measurements

Blood samples were taken from baboon recipients prior to xenotransplantation, regularly during each experiment and before euthanasia. Lactate measurements were performed with Siemens RAPIDLab^®^ 1200 Systems (Siemens, Munich, Germany). The measurements before the beginning of surgical procedures on the day of xenotransplantation were defined as baseline (Pre XTx).

### 2.5. Measurement of Endothelial Glycocalyx Components

The concentrations of the shedded endothelial glycocalyx components in the plasma samples were analyzed using the following enzyme-linked immunosorbent assays: Human CD138 ELISA Kit (Diaclone SAS, Besançon, France) detects natural and recombinant human Syndecan-1 protein without cross reactivity with other human soluble molecules; the HS ELISA Kit (Wuhan Fine Biotech Co., Ltd., Wuhan, China) specifically recognizes heparan sulfate with no obvious cross reaction with other analogues, according to the manufacturer; the Hyaluronan Enzyme-Linked Immunosorbent Assay Kit (Echelon Biosciences Inc., Salt Lake City, UT, USA) detects HA molecules that are as small as 6.4 kDa [39]. ELISAs were performed according to the manufacturer’s protocol. The intra- and inter-assay variability for each ELISA kit is indicated by the coefficient of variation, as provided by the manufacturer: HS: 5.2%; 5.3%, HA: <20%; <10%, and Syndecan-1: 6.2%; 10.2%.

### 2.6. Statistics

Data collection and analyses were performed with Excel 2019 (Microsoft, Redmond, WA, USA) and GraphPad Prism 9.0 (GraphPad Software Inc., Boston, MA, USA). Data are presented either as single measurements or as group means ± SD if not indicated otherwise.

## 3. Results

We present analyses of the three endothelial glycocalyx components—the two glycosaminoglycans, hyaluronan and heparan sulfate, as well as the proteoglycan syndecan-1—in the perioperative (n = 4) and postoperative period (n = 5) after pig-to-baboon orthotopic cardiac xenotransplantation experiments. Other data from these experiments, e.g., pre- and postoperative immunologic parameters, causes of death and myocardial histological findings have not been subject to this retrospective data analysis and have been published in detail elsewhere [4,5,14]. Some of these data are summarized in Table 1.

Two experiments, #17186 and #17290 (group I), were deliberately terminated when the predetermined period of 90 postoperative days (set by the regulatory authorities) was reached, with the animals in excellent clinical condition [4,5]. Two animals, #17494 and #17492 (group II), were tested positive for PCMV/PRV [14] and experiments were terminated after 15 and 27 days, respectively, because they presented with signs of multiorgan failure [5]. In contrast to the other animals, baboon #16755 received a heart that was ischemically preserved and no growth inhibitory drugs were administered. This animal developed progressive diastolic left ventricular failure because of myocardial hypertrophy and associated terminal liver disease [4].

### 3.1. Baseline Values of the Circulating Plasma Endothelial Glycocalyx Components

**Figure 1:**
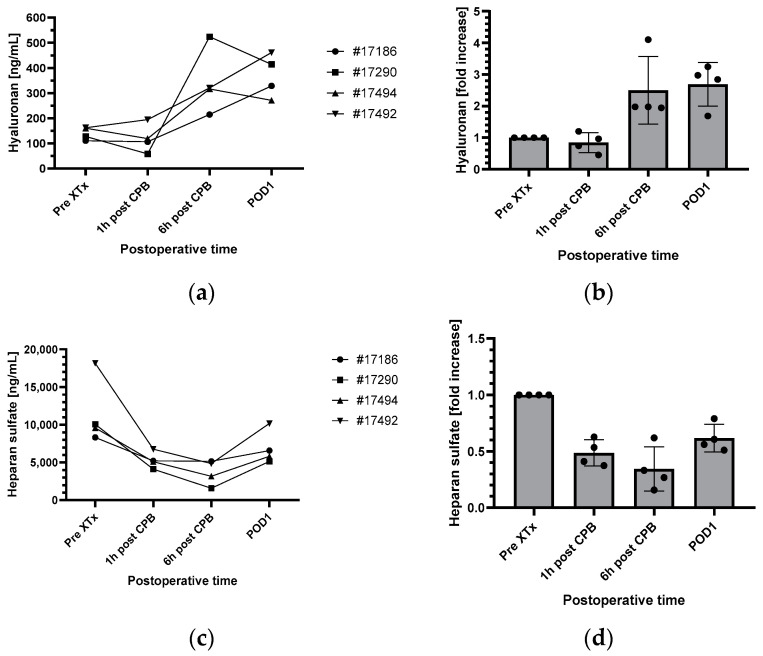

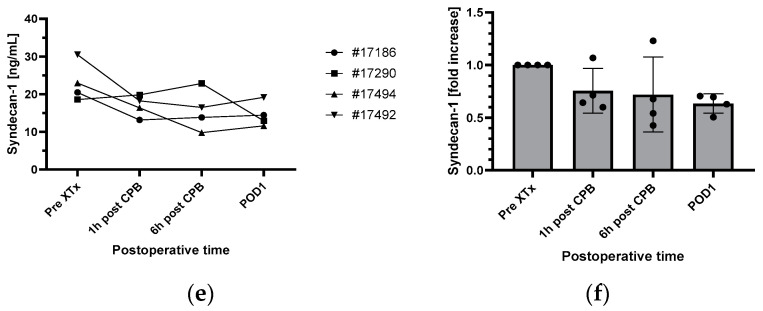
Perioperative changes of hyaluronan (**a**,**b**), heparan sulfate (**c**,**d**) and syndecan-1 (**e**,**f**) in absolute values (ng/mL) and as fold increases compared to the preoperative values (left and right, respectively). (**b**,**d**,**f**) Mean values ± SD (n = 4). CPB, cardiopulmonary bypass; POD1, first postoperative day; XTx, xenotransplantation.

Baseline hyaluronan levels (Pre XTx) were 140.197 ± 24.430 ng/mL, baseline heparan sulfate levels were 11,534.683 ± 4480.468 ng/mL and baseline syndecan-1 levels were 23.117 ± 5.228 ng/mL (Figure 1a,c,e).

### 3.2. Perioperative Changes of the Endothelial Glycocalyx Components

In the perioperative period, levels of hyaluronan, heparan sulfate and syndecan-1 showed a consistent course in all four baboons (Figure 1a,c,e).

Hyaluronan levels showed no relevant change 1 h after cardiopulmonary bypass (CPB) was stopped as compared to the baseline levels. When measured 6 h after termination of CPB, hyaluronan levels increased 2.5-fold as compared to the baseline levels and stayed at these increased levels during the first postoperative day (Figure 1a,b).

Heparan sulfate and syndecan-1 decreased in the perioperative period (Figure 1c–f). Compared to their baseline levels, heparan sulfate presented with a decrease around of 0.5-fold at 1 h and 6 h after CPB and during the first postoperative day (Figure 1c,d). The decrease in syndecan-1 levels was less pronounced, compared to heparan sulfate (Figure 1e,f).

### 3.3. Perioperative Lactate Changes and Correlation with Endothelial Glycocalyx Components

**Figure 2:**
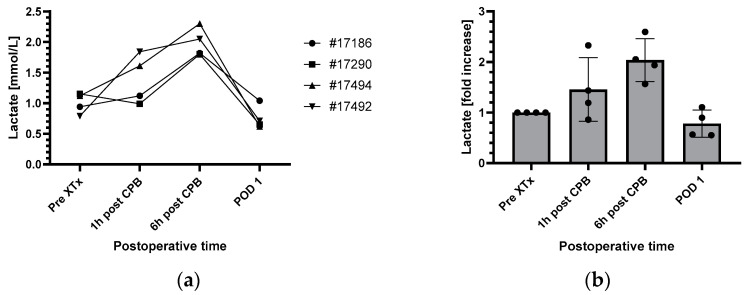

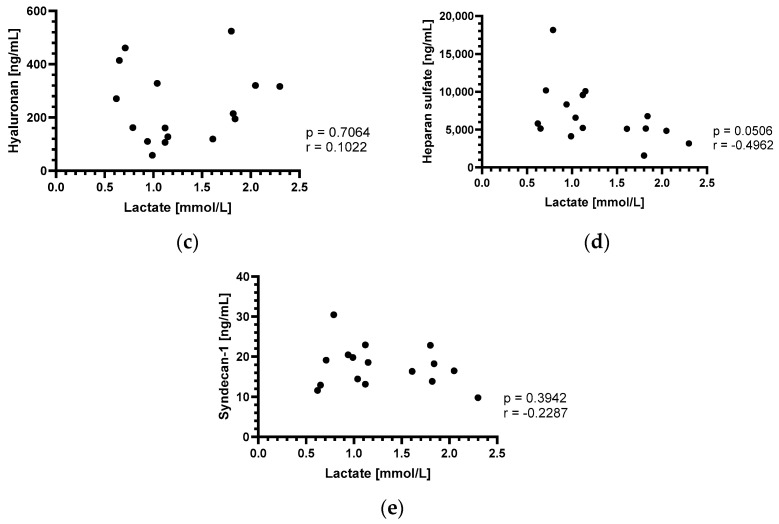
Perioperative courses of serum lactate in absolute values (ng/mL) and as fold increases compared to start of surgery (**a**,**b**). Correlation between perioperative lactate levels and changes of hyaluronan (**c**), heparan sulfate (**d**) and syndecan-1 (**e**). (**b**), mean values ± SD (n = 4). CPB, cardiopulmonary bypass; POD1, first postoperative day; XTx, xenotransplantation.

Similar to the endothelial glycocalyx components, perioperative lactate levels also showed a consistent course in all four baboons (Figure 2a,b). Starting with absolute values around of 1.0 mmol/L at the beginning of surgery, lactate levels increased about 1.5-fold 1 h and about 2-fold 6 h after CPB was stopped. At the first postoperative day, the levels returned to baseline or even lower. There was no significant correlation between the perioperative lactate levels and the levels of hyaluronan (Figure 2c), heparan sulfate (Figure 2d) or syndecan-1 (Figure 2e).

### 3.4. Postoperative Changes of the Endothelial Glycocalyx Components

**Figure 3:**
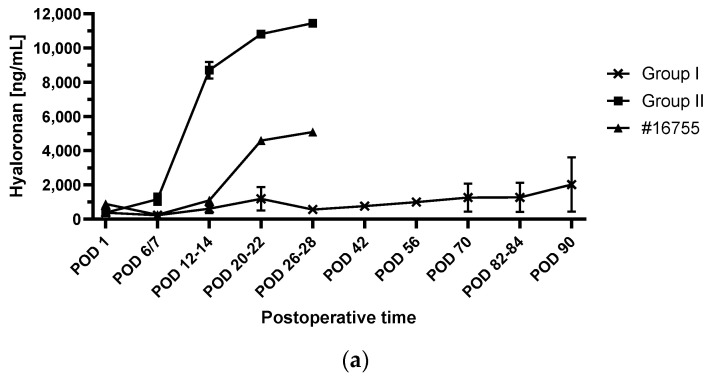

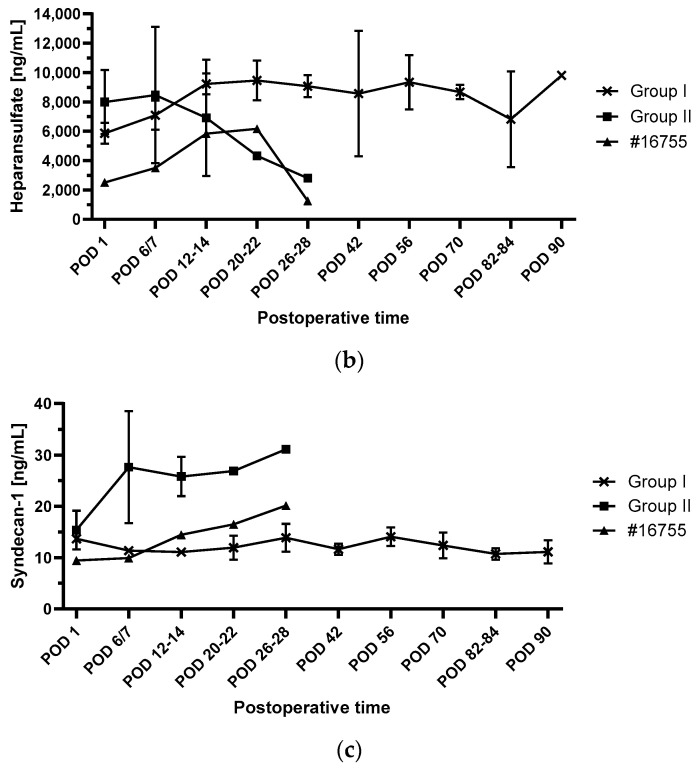
Postoperative courses of hyaluronan (**a**), heparan sulfate (**b**) and syndecan-1 (**c**) plasma concentrations. Group I, experiments deliberately terminated after 90 postoperative days with the baboons in excellent clinical condition, mean values ± SEM (n = 2); Group II, baboons with PCMV/PRV infections, mean values ± SEM (n = 2). PCMV/PRV, porcine cytomegalovirus/roseolovirus; POD, postoperative day.

In contrast to the consistent courses in the perioperative period, postoperative changes of the glycocalyx components were different in the animals whichwere deliberately terminated after 90 postoperative days with the baboons in excellent clinical condition [4] (group I) compared to the baboons with PCMV/PRV infections [5] (group II) (Figure 3).

In the group I animals, all glycocalyx components showed a stable postoperative course without relevant increases or decreases until the end of the experiments after 90 postoperative days (Figure 3a–c).

In contrast to group I, in group II animals, hyaluronan levels showed a slight increase in the first postoperative week followed by a sharp increase in the second postoperative week to values over 10,000 ng/mL, representing about a 100-fold increase compared to the baseline levels (Figure 3a). In group II, heparan sulfate was stable in the first postoperative week and then decreased to levels of around 3000 ng/mL until the end of the experiments (Figure 3b). Syndecan-1 levels presented with a sharp increase in the first postoperative week to levels of around 30 ng/mL and remained at these levels until the end of the group II experiments (Figure 3c).

In baboon #16755, hyaluronan levels were stable until the end of the second postoperative week. Afterwards, hyaluronan increased to values of around 5000 ng/mL until the end of the experiment (Figure 3a). Heparan sulfate ranges were lower as compared to group I and group II, with values of around 3000 ng/mL in the first postoperative days. Within three postoperative weeks, heparan sulfate increased to 6000 ng/mL, followed by a sharp decrease until the end of the experiment (Figure 3b). Syndecan-1 was stable in the first postoperative week and then increased to levels of around 20 ng/mL until the end of the experiment (Figure 3c).

## 4. Discussion

### 4.1. Baseline Values of the Circulating Plasma Endothelial Glycocalyx Components

The hyaluronan and syndecan-1 baseline values in the current study group are comparable to the human setting. In healthy humans, circulating median concentrations of 126.0 ng/mL were described for hyaluronan [40,41] and 29.5 ng/mL for syndecan-1 [41,42], when measured with the same ELISA used in the current study. However, the heparan sulfate baseline level of 11,534.7 ng/mL in the current study is slightly higher than the average values described in three human studies that reported mean concentrations of 4800, 5590 and 7000 ng/mL respectively [43,44,45]. Importantly, in these studies, healthy human individuals showed a great heterogeneity in plasma heparan sulfate concentrations [43,44,45,46]. The higher baseline heparan sulfate levels could be partly due to the strikingly higher baseline value of 18,164.2 ng/mL in animal #17492. Furthermore, it should also be considered that a different heparan sulfate ELISA was used in our study, in comparison to the human study mentioned above.

### 4.2. Perioperative Changes of the Endothelial Glycocalyx Components

Several human studies showed an increase of the circulating glycocalyx components hyaluronan, heparan sulfate and syndecan-1 in adult and infant patients undergoing cardiac [40,41,47,48,49,50] and major vascular surgery [44]. For example, in a human cardiac surgery study, hyaluronan ranges increased about 5-fold when CPB was stopped (a timepoint not available in the current study) and were still about 2-fold higher at 1 h after termination of CPB [40]. In another study in humans undergoing cardiac surgery, hyaluronan levels increased about 1.5-fold at 1 h and about 1.3-fold at 6 h after CPB was stopped and returned to their preoperative levels on the second postoperative day [48]. As the time points assessed were not identical, these human data cannot be directly compared with our analyses. However, it appears as if the perioperative hyaluronan changes in the current group occurred with a time delay compared to the human data—while they showed no relevant change 1 h after CPB in the current study group, hyaluronan levels increased about 2.5-fold at 6 h after CPB was stopped and on the first postoperative day.

In contrast to the human data mentioned above [40,44,47,48], where heparan sulfate levels also increased significantly in the perioperative period, heparan sulfate decreased about 0.5-fold in the current study group at 1 h and 6 h after CPB and on the first postoperative day. This was also seen in another human study during early reperfusion in patients undergoing cardiac surgery [50]. In this study, heparan sulfate decreased by 14% during the first minute after aortic declamping and thereafter remained below the pre-reperfusion level [50]. A decrease of heparan sulfate was also seen in human liver allotransplantation after restoration of the splanchnic and lower body circulation [31]. Heparan sulfate and other glycosaminoglycans adhere rapidly to the damaged glycocalyx [51,52,53], which could explain a decrease of circulating heparan sulfate levels. Furthermore, in human hemorrhage, endogenous glycocalyx preservation coincided with a decrease in circulating heparan sulfate [54]. We therefore assume that the heparan sulfate decrease in the current study could have been caused by rapid endogenous restoration of the glycocalyx. As the recipient baboons all received protamine after CPB, the decrease of heparan sulfate could also be explained by removal of protamine-bound heparan sulfate from the circulation [55].

Although, to our knowledge, there are no human data in this regard, we assume that the perioperative decrease of syndecan-1 in the current study was caused by similar mechanisms as the decrease of heparan sulfate.

Summarizing our perioperative findings, we suggest that there was less glycocalyx shedding in the current study group than in comparable human cardiac surgery studies. In the human setting, the shedding of the endothelial glycocalyx is mainly attributed to ischemia/reperfusion injury [56,57,58], inflammation induced by TNFα [59], the release of atrial natriuretic peptides [40,60] and is furthermore seen as general phenomenon after CPB [48,61]. In all four animals of the perioperative study group, cold non-ischemic heart preservation with continuous perfusion was applied [10]. As this prevents ischemia/reperfusion injury [10], the non-ischemic preservation could explain the reduced glycocalyx shedding in the current study group. In addition to avoiding ischemia/reperfusion injury, the preservation solution containing albumin [4,9,10] could be an important factor in this regard. Addition of albumin to the preservation solution improved endothelial integrity and heart performance in guinea pigs, which was partly explained by the protective effects of albumin on the endothelial glycocalyx [62]. Furthermore, the anesthetic agent sevoflurane, which has proved protective to the endothelial glycocalyx [63,64,65], was used in all animals.

However, with only four animals, the present analysis can only provide initial insights and indications regarding glycocalyx shedding in cardiac xenotransplantation. Further studies with larger numbers of cases and possibly further analyses are needed to answer these questions in more detail.

### 4.3. Perioperative Lactate Changes and Correlation with Endothelial Glycocalyx Components

The changes in perioperative lactate levels in the current study group were comparable to existing human data [48]. In humans, during CPB in cardiac surgery, lactate levels as a parameter of the microcirculation also showed about a 2-fold increase in the perioperative period, which correlated significantly with perioperative syndecan-1 changes [48]. This correlation could be explained by microcirculatory perfusion disturbances caused by the perturbation of the endothelial glycocalyx [56,66,67]. There was no correlation between perioperative lactate changes and changes of hyaluronan, heparan sulfate or syndecan-1 in the current study group, although lactate changes were comparable to the human study mentioned above [48]. This could be explained by less perioperative shedding of the endothelial glycocalyx in the current study group (see above). However, it is also possible that the study group was too small to produce such a correlation.

### 4.4. Postoperative Changes of the Endothelial Glycocalyx Components

There was no relevant change in the levels of hyaluronan, heparan sulfate or syndecan-1 in the two experiments of group I. At best, there was a slight increase in hyaluronan towards the end of the experiments. Both animals showed an unremarkable clinical course without any signs of rejection and were deliberately terminated after 90 days [4,5]. We therefore interpret the current data as a sign that there is no relevant damage to the endothelial glycocalyx after pig-to-baboon cardiac xenotransplantation using current strategies of CD40/CD40L based immunosuppression [8], organ preservation [9,10] and growth inhibition [4]. Furthermore, both baboons in group I received an IL-6 receptor antagonist, a TNFα inhibitor and a C1 esterase inhibitor as part of the anti-inflammatory regimen [4,19]. In humans, the IL-6 receptor antagonist tocilizumab improved the endothelial glycocalyx in rheumatoid arthritis patients [68] and application of a TNFα inhibitor protected against endotoxin-induced endothelial glycocalyx perturbation [69]. Regarding potential beneficial effects of the C1 esterase inhibitor, in vitro data from genetically modified porcine endothelial cells suggest that protection against complement activation contributes to maintaining an intact endothelial glycocalyx [34]. The slight increase in hyaluronan levels in group I animals could be explained by recurring (bacterial) infections [70] treated with antibiotics.

The animals in group II both were tested positive for PCMV/PRV [14,15] and had to be euthanized because of multiorgan failure after 15 and 27 postoperative days, respectively [5]. In these animals, hyaluronan and syndecan-1 levels showed a marked increase, whereas heparan sulfate levels decreased until the end of the experiments. We assume that these changes were caused, at least in part, by infection with PCMV/PRV. To our knowledge, there are so far no data on the interaction of PCMV/PRV with the endothelial glycocalyx. However, there are data on other virus infections leading to damage of the endothelial glycocalyx in humans [71,72,73,74,75], for example COVID-19 [71], H1N1 influenza [72], hanta [75] and dengue virus [73,74]. For example, in H1N1 influenza infections, elevated hyaluronan levels were associated with an increase in mortality rate [76] and increased plasma syndecan-1 levels were an independent risk factor for mortality [77]. In the case of PCMV/PRV, high levels of tissue plasminogen activator and inhibitor 1 complexes in baboons transplanted with a PCMV/PRV-positive pig heart indicate a complete loss of the pro-fibrinolytic properties of the endothelial cells [14]. These findings and the fact that PCMV/PRV does not infect human cells [78] suggest that PCMV/PRV may directly interact with endothelial cells. Furthermore, an increased level of IL-6 was found in PCMV/PRV-positive animals #17494 and #17492 [14]. These increased IL-6 levels may have contributed to the glycocalyx damage seen after infection by the influenza virus H1N1 [72]. Proinflammatory cytokines, such as IL-6, are known to activate enzymes named sheddases, which induce glycocalyx degradation [79,80].

The fifth animal in the postoperative study group, #16755, had to be euthanized because of heart and liver failure due to myocardial overgrowth after 30 postoperative days [4]. Comparable to group II, hyaluronan and syndecan-1 increased and heparan sulfate decreased, thus indicating damage to the endothelial glycocalyx. As the donor heart was ischemically preserved in this animal, this could have been caused by ischemia/reperfusion injury [56,57,58]. However, we assume that the increase should already have been noticeable on the first postoperative day, if ischemia/reperfusion injury was the cause. Since this was not the case, we assume that the changes in the endothelial glycocalyx were caused by the complications of myocardial overgrowth of the xenograft [4,11]. Currently, there are no data on glycocalyx degradation and cardiac xenograft overgrowth. However, in human patients with heart failure of other entities damage to the glycocalyx has been described [81,82].

### 4.5. Limitations

The number of experimental animals in the current retrospective analysis is limited and there is no “classical” control group. Therefore, the available data do for example not allow for any further statistical analyses. However, considering the principles of the 3 Rs [83], the high value of donor pigs and recipient baboons, and the complexity of the experiments, we believe these data are worth publishing and adequate for gaining first insights into the endothelial glycocalyx in pig-to-baboon cardiac xenotransplantation.

We investigated glycocalyx injury based on the measurement of circulating glycocalyx components in the plasma. Since it is still a matter of debate whether these soluble glycocalyx components correlate adequately with glycocalyx structure and function [84], the plasma concentrations of hyaluronan, heparan sulfate and syndecan-1 presented here can only be interpreted as surrogate biomarkers for glycocalyx integrity.

Further, ideally, prospective studies with more experiments and possibly additional analyses should be performed to obtain a deeper understanding of the endothelial glycocalyx in cardiac xenotransplantation.

## 5. Conclusions

The current analysis provides first insights into changes of the endothelial glycocalyx after pig-to-baboon cardiac xenotransplantation. Using current strategies of cardiac xenotransplantation, damage to the endothelial glycocalyx seems to be comparable or even less pronounced than in similar human settings. At the same time, the data from experiments where current strategies, such as PCMV/PRV elimination, non-ischemic preservation and growth inhibition, could not be applied indicate that damage to the endothelial glycocalyx also plays an important role in cardiac xenotransplantation.

## Figures and Tables

**Table 1:** Overview of the study group. F, female; M, male; PCMV/PRV, porcine cytomegalovirus/porcine roseolovirus.

Experiment	DPZ-ID	Donor		Recipient		Group	Survival	Growth Inhibition	Preservation	Causes for Euthanasia
		Sex	Weight	Sex	Weight					
1	#16755	M	15.8 kg	M	16.0 kg	-	30 days	No	Ischemic	Heart and liver failure [4]
2	#17186	F	19.3 kg	M	21.5 kg	I	90 days	Yes	Non-ischemic	Study endpoint [4,5]
3	#17290	F	12.7 kg	M	13.7 kg	I	90 days	Yes	Non-ischemic	Study endpoint [4,5]
4	#17494	M	11.6 kg	M	16.0 kg	II	15 days	Yes	Non-ischemic	Multiorgan failure (PCMV/PRV) [5,14]
5	#17492	F	24.0 kg	M	26.0 kg	II	27 days	Yes	Non-ischemic	Multiorgan failure (PCMV/PRV) [5,14]

## Data Availability

The raw data supporting the conclusions of this article will be made available by the authors on request.
